# The Key Role of Peltate Glandular Trichomes in Symbiota Comprising Clavicipitaceous Fungi of the Genus *Periglandula* and Their Host Plants

**DOI:** 10.3390/toxins7041355

**Published:** 2015-04-16

**Authors:** Ulrike Steiner, Sabine Hellwig, Mahalia A. Ahimsa-Müller, Nicola Grundmann, Shu-Ming Li, Christel Drewke, Eckhard Leistner

**Affiliations:** 1 Institut für Nutzpflanzenwissenschaften und Ressourcenschutz, Universität Bonn, Nußallee 9, D-53115 Bonn, Germany; E-Mail: u-steiner@uni-bonn.de; 2 Institut für Pharmazeutische Biologie, Universität Bonn, Nußallee 6, D-53115 Bonn, Germany; E-Mails: s.hellwig@gmx.eu (S.H.K.); mahimsa@yahoo.com (M.A.A.-M.); Cdrewke@uni-bonn.de (C.D.); 3 Institut für Pharmazeutische Biologie und Biotechnologie, Universität Marburg, Deutschhausstraße 17 1/2, D-35037 Marburg, Germany; E-Mails: grundmannni@gmx.de (N.G.); shuming.li@staff.uni-marburg.de (S.-M.L.)

**Keywords:** sesquiterpenes, ergot alkaloids, peltate glandular trichomes, bidirectional secretion, *Periglandula*, Convolvulaceae

## Abstract

Clavicipitaceous fungi producing ergot alkaloids were recently discovered to be epibiotically associated with peltate glandular trichomes of *Ipomoea asarifolia* and *Turbina corymbosa*, dicotyledonous plants of the family Convolvulaceae. Mediators of the close association between fungi and trichomes may be sesquiterpenes, main components in the volatile oil of different convolvulaceous plants. Molecular biological studies and microscopic investigations led to the observation that the trichomes do not only secrete sesquiterpenes and palmitic acid but also seem to absorb ergot alkaloids from the epibiotic fungal species of the genus *Periglandula*. Thus, the trichomes are likely to have a dual and key function in a metabolic dialogue between fungus and host plant.

## 1. Introduction

The structural diversity of natural products reflects the enormous biochemical capabilities of plants and microorganisms [1]. The *raison d’etre* for the efforts that organisms make to produce natural products had not been understood for a long time. In the last couple of years, however, it has become evident that the ecological role natural products play in the attraction of beneficial organisms or deterrence of competitors and pathogens is an advantage to the respective organism which may either synthesize natural products by itself or acquire these products from endo- or epiphytic microorganisms [2,3,4,5]. The basis for the ecological role of natural products is the enormous structural diversity which makes it difficult for competing and attacking organisms to overcome the barrier set by a plant or microorganism sequestering natural products [2,3,4,5].

Among the many different types of natural products, terpenoids are the most abundant group comprising *ca.* 25,000 different structures [1]. Mono- and sesquiterpenes, which are often products of secretory glands, exert their physiological, chemical, and ecological roles as part of blends comprising also fatty acids or derivatives of cinnamic acids or nitrogen- or sulfur-containing compounds [6]. The high vapour pressure at ambient temperature, structural diversity, and occurrence in variable combinations makes mono- and sesquiterpenes perfect conveyors of specific signals between interacting organisms [7]. We suspect that sesquiterpenes may be mediators in a symbiotic system that has recently been described [8,9,10,11].

This system consists of an epibiotic clavicipitaceous fungus, *Periglandula ipomoeeae* and its host plant, *Ipomoea asarifolia* belonging to the family Convolvulaceae (“Morning glories”). A similar association exists between the fungus *Periglandula turbinae* and the convolvulaceous host plant *Turbina corymbosa* [9]. We call the former system *Periglandula*/*Ipomoea* symbiotum, the latter *Periglandula*/*Turbina* symbiotum.

The two fungi are representatives of the newly described genus *Periglandula* belonging to the family Clavicipitaceae [12]. They are seed transmitted and are intimately associated with peltate glandular trichomes on the adaxial leaf surface of their host plants [13]. It is likely that similar associations occur in an estimated 450 plant species belonging to the tribe *Ipomoeae* within the family Convolvulaceae [14,15]. We failed to axenically culture the fungus *Periglandula spp*. However, an *in vitro* experimental system between the clavicipitaceous *P. ipomoeae* fungus and cultivated *I. asarifolia* host plant cells has been described. In this latter system, plant cells and hyphal compartments are co-cultivated in a plant cell culture [8,16]. Fungus and plant cells grow asymptomatically with no hypersensitive response and with both systems apparently keeping each other in check. Remarkably, although fungus and plant cells are both present, the culture consisting of fungal cells and undifferentiated plant cells is devoid of ergot alkaloids. Changing the hormonal regime of the cell culture, however, results in a differentiation of cell lumps, creating a plantlet that carries exclusively the *P. ipomoeae* fungus and ergot alkaloids. This demonstrates that the morphological differentiation of the plant and the fungus and, very likely, the association of the fungus with peltate glandular trichomes are a prerequisite for ergot alkaloid biosynthesis.

Treatment of the plants with fungicides eliminates both the fungus as well as the alkaloids [13]. In contrast, the production of sesquiterpenes is not affected. Thus, the elimination of fungi and alkaloids is a specific process that does not affect secondary metabolism of the symbiotum in general but specifically the development of the alkaloid producing clavicipitaceous *Periglandula* fungus. It also shows that sesquiterpenes are products of the plants but not of the fungus [13,17]. Simultaneous elimination of fungus and alkaloids assigns ergot alkaloid biosynthesis to the *Periglandula* fungus. This is in agreement with the fact that the complete set of genes necessary to synthesize ergot alkaloids is located within the *P. ipomoeae* fungus [17,18].

The pivotal step in alkaloid biosynthesis is the prenylation of tryptophan with dimethylallyl pyrophosphate catalyzed by 4-(γ,γ-dimethylallyl)tryptophan synthase [17]. This enzyme is encoded by the *dmaW* gene which is part of the fungal ergot alkaloid gene cluster present within *P. ipomoeae* and *P. turbinae.* The *dmaW* gene from *P. ipomoeae* was overexpressed. Substrate specificity and kinetic data leave no doubt as to its role in ergot alkaloid biosynthesis [17]. One may therefore conclude that ergot alkaloids, and possibly also their biosynthesis, reside within the fungus. On the other hand, it is generally accepted that ergot alkaloids, which are well known constituents of clavicipitaceous fungi, confer environmental tolerance, fitness, insecticidal activity, and feeding deterrence to their plant hosts [19,20,21,22,23,24]. If indeed the ergot alkaloids are a benefit to the plant, they should be located in the plant but not in the fungus. Ultrasonic treatment of *I. asarifolia* leaves removes the fungus and allows for the separate analysis of both symbiotic organisms. This shows that 95% of the alkaloids present in the symbiotic leaves reside in the plant organ but not in the associated hyphae [11,17].

Therefore, we postulate that the biosynthesis of ergot alkaloids takes place in the fungus and that a transport of ergot alkaloids occurs from the fungal *Periglandula* hyphae to the leaves of the host plants *I. asarifolia* and *T. corymbosa.* This is of interest because the fungal hyphae are known to be intimately associated with peltate and volatile oil secreting glands on the leaf surface but do not seem to penetrate the leaf epidermis [16,24]. Thus, the glandular trichomes may have a dual function as they do not only secrete volatile oil but are likely to absorb and transport ergot alkaloids from the fungal hyphae into the plant leaves. The present article investigates this hypothesis and summarizes observations which are in favour of this idea.

## 2. Results

### 2.1. The Oil Secreting Function of Peltate Glandular Trichomes Present on the Adaxial Leaf Surface of Convolvulaceous Plants

**Figure 1:**
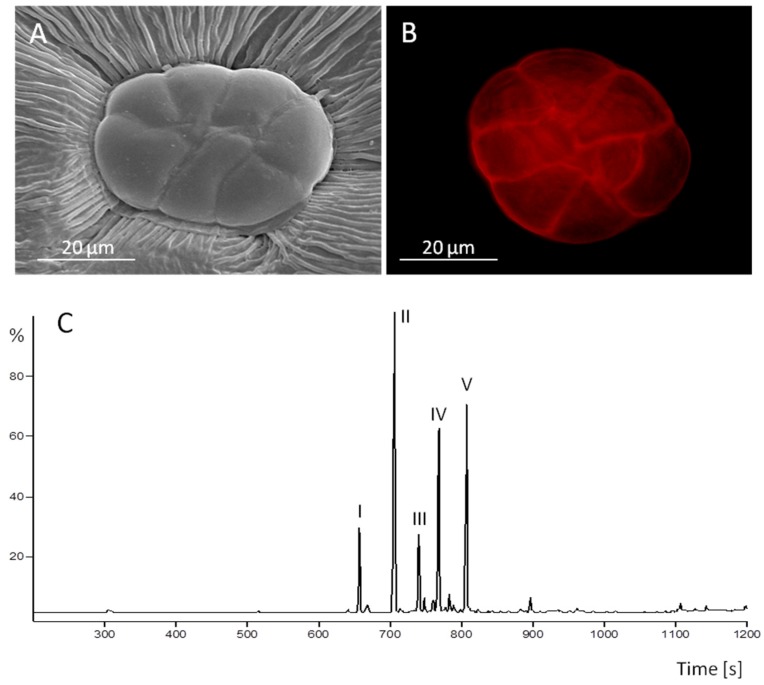
Oil secreting function of peltate glandular trichomes present on the adaxial leaf surface of *I. asarifolia*: (**A**) a gland consists of up to eight glandular secretory cells (REM); (**B**) staining with Nile red detects lipophilic compounds in the subcuticular oil storage cavity above the secretory cells (fluorescence microscopy); (**C**) constituents in the oil compartments are sesquiterpenes α-copaene (I), (*E*)-β-caryophyllene (II), α-humulene (III), germacrene (IV), and δ-cadinene (V) as analyzed by gas chromatography. After fungicide treatment glands shown in **A** and **B** are devoid of hyphae. Components in **C** were analyzed before fungicide treatment.

The symbiotic systems described here are characterized by two classes of natural products, ergot alkaloids, and lipophilic compounds, comprising sesquiterpenes and a fatty acid. The possible role of the terpenoids in the interaction between fungi and convolvulaceous plants is still unexplored, as the elusive fungi are hitherto non cultivable under axenic condition. It is possible, however, that the oil components are mediators of the interaction between the oil producing peltate glandular trichomes (Figure 1A,B) and their closely associated clavicipitaceous fungi [11] because sesquiterpenes are not only signalling compounds but are consistently present in the symbiota described in this article. The volatile oils of *I. asarifolia* (white and red blooming varieties) and *T. corymbosa* were isolated by hydrodestillation. The volatile oil components of the white blooming *I. asarifolia* and the *T. corymbosa* plants were identified using gas chromatography, mass spectra, and restriction indices (Figure 1C) [25]. The volatile oil components of the red blooming *I. asarifolia* plant were tentatively identified by comparison with published mass spectra [25,26]. *I. asarifolia* (white blooming) produces the sesquiterpenes α-copaene, (*E*)-β-caryophyllene, α-humulene, germacrene, and δ-cadinene (Figure 1) while sesquiterpenes theaspirane isomer I and II as well as damascenone and bergamotene are products of *T. corymbosa*. The red blooming *I. asarifolia* plant produces the following tentatively identified sesquiterpenes, dihydroedulane, α-cubebene, (*E*)-β-damascenone, α-copaene, β-bourbonene, (*E*)-β-caryophyllene, cadina-3,5-diene, α-humulene, *cis*-muurola-4,5-diene, γ-muurulene, germacrene d, bicyclosesquiphellandrene, γ-amorphene, bicyclogermacrene, γ-cadinene, δ-cadinene, zonarene, and α-corocalene. Although the white and the red blooming *I. asarifolia* plants represent the same species they produce different sets of sesquiterpenes.

In all three experimental systems described above, sesquiterpenes are components within the volatile oil as demonstrated in Figure 1C for *I. asarifolia* (white blooming). As expected [6], the oil of all three species tested contains also at least one non-terpenoid compound, hexadecanoic (*i.e.*, palmitic) acid [26].

### 2.2. Ergot Alkaloid Biosynthesis in the Fungal Mycelium

The alkaloids isolated from the *Periglandula/Ipomoea* symbiotum are chanoclavine-I, ergonovine, the two isoforms of lysergic acid-α-hydroxy ethylamide, the two lysergic acid amide isoforms, agroclavine and ergobalansine/ergobalansinine [8,9,11,15,27,28]. Due to a lack of reference material, the two isomers ergobalansine and ergobalansinine could not be identified in our hands. We observed the same ergot alkaloids present in the *Periglandula*/*Ipomoea* symbiotum also in the *Periglandula*/*Turbina* symbiotum. In addition, the latter symbiotum contains elymoclavine [8,9]. The ergobalansine isomers were hitherto not reported from *T. corymbosa*.

**Figure 2:**
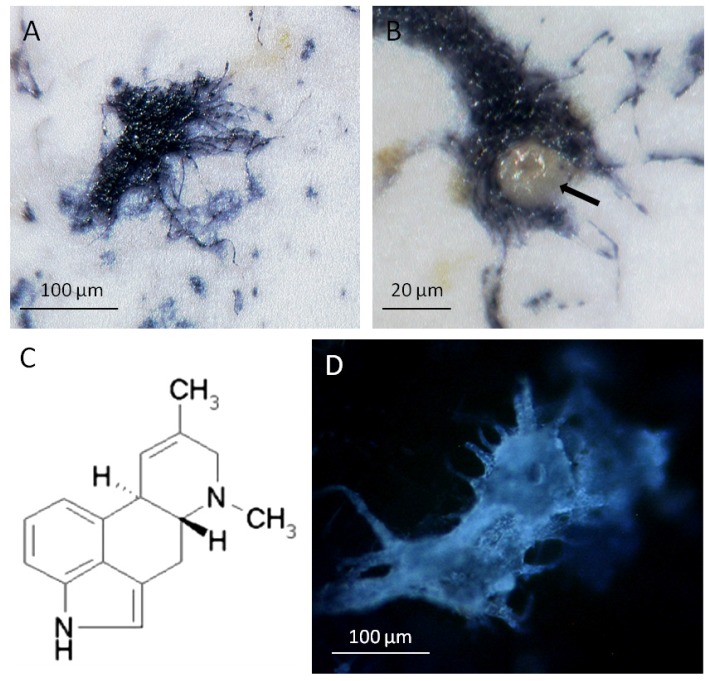
Ergot alkaloid biosynthesis in the fungal mycelium: (**A**) hyphae of *P. ipomoeae* developed on the leaf surface of *I. asarifolia* stained blue after binding with a polyclonal antibody directed to the 4-(γ,γ-dimethylallyl)tryptophan synthase (DmaW enzyme); (**B**) a peltate glandular trichome surrounded by the fungus showed no labelling (arrow); (**C**) agroclavine, an alkaloid of the ergot biosynthetic pathway. A trace of this alkaloid is the only alkaloid detectable in the hyphae of *P. ipomoeae* at the late stages of leaf development [11]. At this time, the bulk of ergot alkaloids are located within the leaves of *I. asarifolia*; (**D**) at an early stage of leaf development, alkaloids are visualized by their UV-autofluorescence within the mycelium. This fluorescence faded away during leaf expansion.

The pivotal step in ergoline alkaloid biosynthesis is catalyzed by the 4-(γ,γ-dimethylallyl)tryptophan synthase (DmaW) [17,29,30,31]. The enzyme is encoded by the *dmaW* gene within *P. turbinae* (accession No DQ121454) and *P. ipomoeae* (accession No DQ 121453) [17]. The experimental approach for the sequencing of the *dmaW* gene involved PCR and chromosome walking [17] and, in addition, reverse genetic experiments based on the isolation of mRNA [17,32] transcribed from the *dmaW* genes present in *P. ipomoeae* and *P. turbinae*. The genomic DNA of the *dmaW* gene consists of 1486 base pairs (*P. ipomoeae*) or 1483 bp (*P. turbinae*), respectively, whereas the cDNA comprises 1371 bp in both cases. There are two introns with a length of 58 bp (*P. ipomoeae*, intron 1) or 55 bp (*P. turbinae*, intron 1) and 57 bp for intron 2 in both cases. The presence of the mRNA transcribed from the *dmaW* gene in both cases allows us to conclude that the *dmaW* gene is not only present in both *Periglandula* species but also subject to transcription in both fungi. The same gene is not detectable in the respective host plants *I. asarifolia* and *T. corymbosa* [17].

The fungal hyphae on the leaf surface were stained by binding to a polyclonal antibody directed to the DmaW enzyme (Figure 2A,B). In contrast, the glandular trichomes surrounded by the fungal mycelium showed no reaction (Figure 2B). It follows that the DmaW enzyme is present within the fungal hyphae, indicating that not only transcription but also translation take place within the fungus rather than the plant cells. This is in agreement with the presence of agroclavine in the symbiotic fungus (Figure 2C). Indeed, the entire steps in ergot alkaloid biosynthesis are likely to take place within the fungus because the complete set of ergot biosynthetic genes is present within the fungus [18]. At the early stage of leaf development, alkaloids can be visualized by their blue UV-autofluorescence within cells according to Mulac *et al.* [33] (Figure 2D). On mature leaves, the UV-autofluorescence faded away from the hyphae. This coincides with the observation that only a hardly detectable trace of agroclavine [11] remains as a constituent of the fungal hyphae. Simultaneously, 95% of fungal alkaloids of the whole symbiotum are found within the leaves [17].

### 2.3. Peltate Glandular Trichomes of Convolvulaceous Plants and Periglandula Hyphae are Part of a Functional Entity

#### 2.3.1. Kinetic Experiments

**Figure 3:**
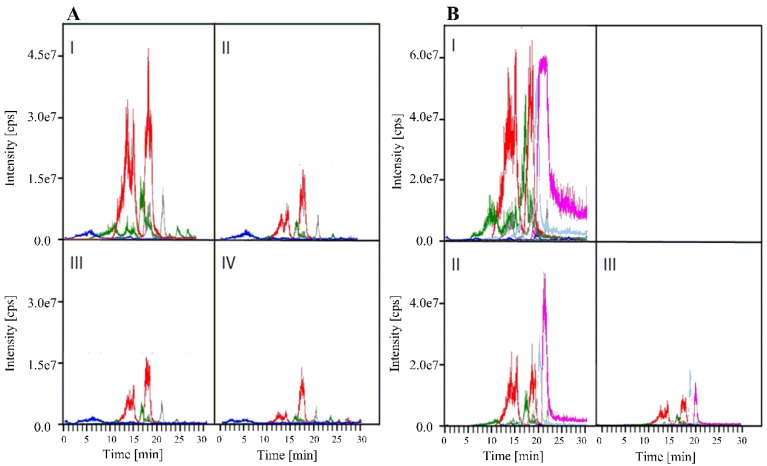
Multi-ion HPLC/MS/MS chromatography of the alkaloid fraction of leaves at different developmental stages of the *Periglandula*/*Ipomoea* (**A**) symbiotum, *i.e.*, leaf buds (I), open buds (II), medium sized leaves (III), fully expanded leaves (IV). Alkaloids are colour coded: chanoclavine-I (blue, 6 min); lysergic acid amide (green,11.0 min); isolysergic acid amide (green, 16.5 min); lysergic acid α-hydroxyethylamide (red, 13.0 min); isolysergic acid α**-**hydroxyethylamide (red, 14.5 min), ergonovine (grey, 21 min); (**B**) *Periglandula*/*Turbina* symbiotum, *i.e.*, leaf buds (I), medium sized leaves (II), fully expanded leaves (III). Alkaloids are colour coded: lysergic acid amide (green, 10.5 min); isolysergic acid amide (green, 17.0 min); lysergic acid α**-**hydroxyethylamide (red, 13.0 min), isolysergic acid α-hydroxyethylamide (red, 14.5 min); ergonovine (grey, 21.5 min), elymoclavine (light blue, 15.5 min), agroclavine (margenta, 21 min). In each case, 9 g (wet weight) of leaf material was analyzed under identical conditions. The identity of alkaloids was also checked by comparison with authentic compounds.

The identity of the ergot alkaloids were confirmed by using a HPLC/MS/MS system and the quantity determined as dependant on the leaf development. The result is shown in Figure 3 in the form of multi-ion chromatograms. Apparently, alkaloid biosynthesis takes place at a very early time of leaf development. Quantitative estimation of alkaloids (Figure 3 and Table 1) reveals that, on a fresh weight basis, leaf buds contain the highest amount of ergot alkaloids that declines by leaf expansion. In contrast, the composition of the alkaloidal fraction does not change with the age of the developing leaf (Figure 3).

The relation between the amount of ergot alkaloids in the two symbiota, fungal colonisation of the trichomes, and number of peltate glandular trichomes per leaf area as influenced by the leaf development are shown in Table 1. The fungal colonisation was quantitated by determination of ergosterol, a typical fungal metabolite [34]. The amount of trichomes per square mm was counted using a microscope. Once again, the experiments were carried out on both *I. asarifolia* and *T. corymbosa* plants. The highest concentration of ergot alkaloids coincides with the highest density of fungal colonisation and the highest number of trichomes per leaf area as observed in leaf buds of both plant species. During leaf development, not only alkaloid content (Figure 3, Table 1) but all three parameters (amount of ergot alkaloids, ergosterol, and number of glandular trichomes) are “diluted” by leaf expansion and drop on a fresh weight basis to *ca*. 10 percent or even less in a mature leaf (compare Table 1).

**Table 1:** Relation between developmental stage of leaves (determined by length of the rhachis), amount of ergosterol extracted from leaves (as a measure of the fungal colonisation), content of ergoline alkaloids (given as ergonovine maleate equivalents), and number of peltate glandular trichomes per square millimeter present on the adaxial leaf surface of *I. asarifolia* (A) and *T. corymbosa* (B).

Stage	Leaves	Ergosterol		Ergoline alkaloids		Glandular trichomes	
	Length of Rhachis (cm)	μg (gFW)^−1^	%	μg (gFW)^−1^	%	Number (mm^2^)^−1^	%
A							
Bud	1.0–2.0	13.9	100.0	48.3	100.0	77.0	100.0
Open bud	1.5–2.0	8.3	69.7	15.9	32.9	34.0	44.2
Medium sized leaf	2.5–4.0	0.9	6.5	11.9	24.6	16.5	21.4
Fully expanded leaf	4.5–6.5	1.7	12.2	6.4	13.3	8.3	10.8
B							
Bud	1.5–3.0	13.5	100.0	110.8	100.0	37.8	100.0
Medium sized leaf	3.5–5.0	trace	>0	53.3	48.1	10.6	28.0
Fully expanded leaf	5.5–7.0	trace	>0	9.5	8.6	4.7	12.4

#### 2.3.2. The Glandular Trichomes as Interface of the Plant/Fungus Symbiotum

**Figure 4:**
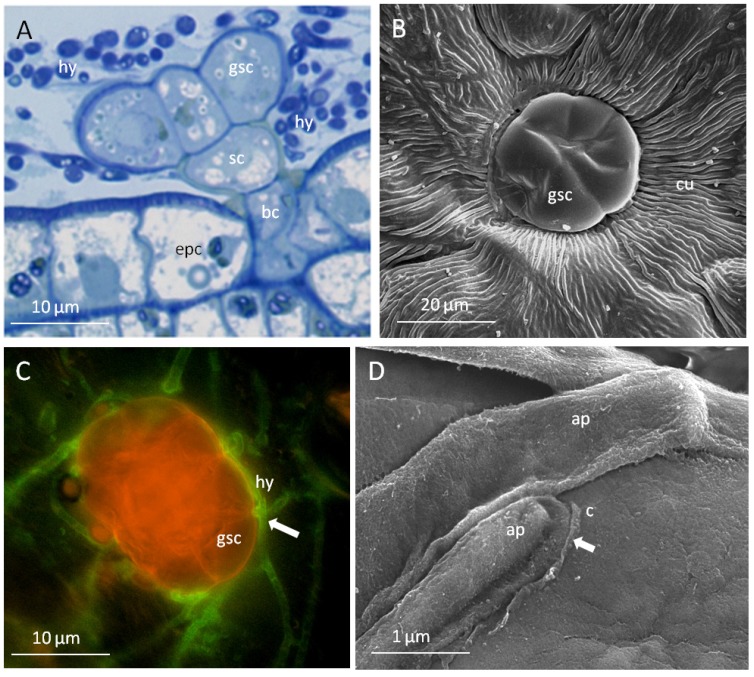
Peltate glandular trichomes as interfaces of the plant/fungus symbiotum *Periglandula*/*Ipomoea*: (**A**) epiphytic colonisation by fungal hyphae (hy) of the glandular trichome composed of a basal cell (bc), stalk cell (sc) and secretory cells (gsc) arising from epidermal cells (epc) (semi thin section, stained with toluidine blue, light microscopy); (**B**) different surface properties of the cuticle of epidermal cells (cu) and secretory cells of trichomes (gsc) (REM); (**C**) adhesion and penetration sites (arrow) of the fungus at the trichome cuticle (hyphae stained with WGA, secretory cells stained with Nile red, fluorescence microscopy); (**D**) formation of appressorium-like structure (ap) on the cuticle (c) of the secretory cells accompanied by degradation of the cuticle (arrow).

**Figure 5:**
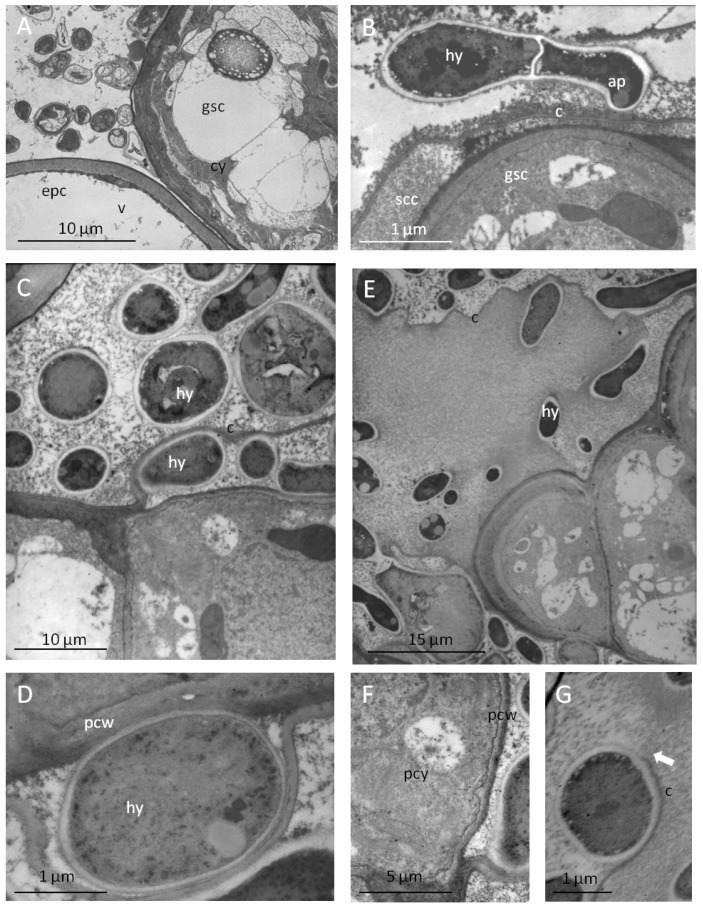
Ultra structural characterization by TEM of contact sites of the plant fungus symbiotum *Periglandula*/*Ipomoea:* (**A**) dense cytoplasm of colonized secretory cells (gsc) in contrast with epidermal cells poor in cytoplasm and completely filled with a central vacuole (v); (**B**) contact site of a hyphae (hy) forming an appressorium-like structure (ap) on the cuticle (c) of the secretory cell covering the subcuticular oil storage cavity; (**C**) hyphae colonising the outer side of the cuticle and the subcuticular cavity after penetration of the cuticle; (**D**) alteration in the structure of the plant cell wall (pcw) at the contact site with the fungus; (**E**) disruption of the cuticle during the colonisation of the subcuticular oil storage cavity; (**F**) accumulation of mitochondria, membrane systems, and ribosomes as indicators of high metabolic activity at the plant/fungus contact site; (**G**) disrupted cuticle (c) of the glandular secretory cell.

Since ergot alkaloids are synthesized in fungi but sequestered in the leaf material of the host plant, transport processes shifting ergoline alkaloids into the plant must take place. A close association between fungus and glandular trichomes would be a prerequisite for such a process. The specific type of contact between the mycelium of the fungi and glandular trichomes are shown in Figure 4 and Figure 5.

The peltate glandular trichomes, composed of a basal cell, a stalk cell and secretory cells, are colonised epibiotically by the mycelium of *Periglandula* sp. being concentrated on the cuticle above the subcuticular oil storage cavity (Figure 4A). The cuticle of the trichomes is smooth and thin as opposed to the cuticle covering the epidermal cells which is characterized by typical thick wax ridges (Figure 4B). The thin cuticle above the subcuticular oil storage cavity cells seem to be well suited for the penetration by the fungus (Figure 4C). Accordingly, on the smooth surface of the trichomes appressorium-like structures were formed at the tips of hyphae encircling the glands (Figure 4D and Figure 5B). However, they are different from typical appressoria formed at the tips of germ tubes of spores, which ceases polar growth, hooks, and begins to swell. The formation of spores by *Periglandula* sp. was never observed. 

Cross sections through glandular cells which were covered with hyphae reveal that they are not only located on top but also underneath the cuticle (Figure 5). After degradation of the cuticle by appressorium-like structures which are embedded in a dense matrix, the fungus appears to penetrate the cuticle and thus is able to enter the subcuticular oil storage cavity (Figure 5B,C). There the fungus was in direct contact with the wall of a glandular cell. This cell wall showed a reduced thickness compared to the epidermal cell walls (Figure 5A). At these contact areas, alterations of the cell wall structure occur making them appear less compact and more transmissible (Figure 5D). The modified cell wall is likely to allow nutrients and possibly also alkaloids to move more freely across the wall [35]. 

The secretory cells showed a high density of cell organelles, mitochondria, and ribosomes, indicating their high metabolic activity (Figure 5A,F). Following the penetration, the cuticle is locally dissolved (Figure 5E,G). Consequently, lipophilic oil components are released from the subcuticular cavity and can possibly serve as an easily accessible nutrient source for the epibiotic fungus (compare [36]).

**Figure 6:**
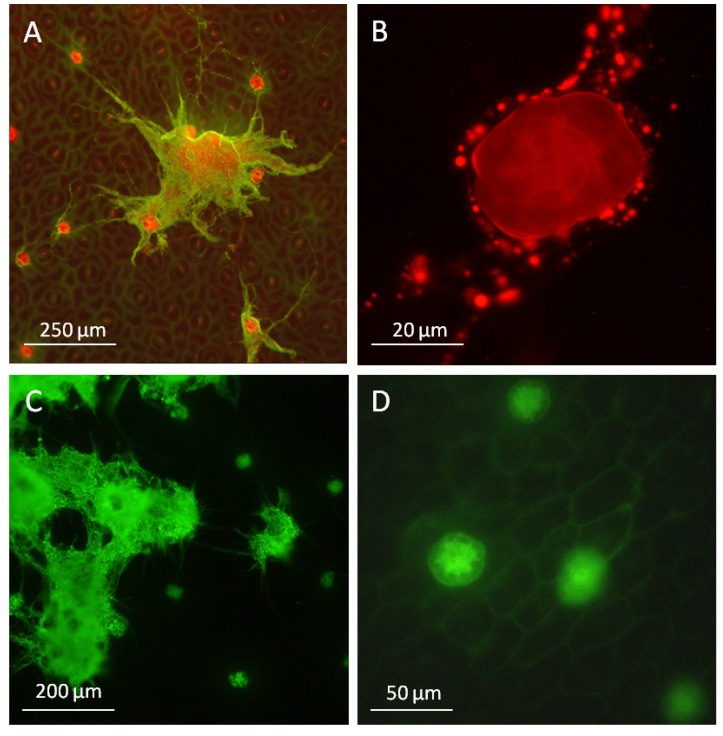
Secretion and uptake of metabolites in the plant fungus symbiotum *Periglandula/Ipomoea* (fluorescence microscopy): (**A**) release of lipids (stained with Nile red) by secretory glandular cells encircled by the fungus; (**B**) lipid vacuoles (stained with Nile red) in hyphae; (**C**) uptake of 6(5)carboxyfluorescein diacetate into the mycelium of the fungus and into glandular trichomes; (**D**) epidermal cells remained unstained, a light staining of mesophyll cells under the glandular trichomes were observed after 30 min incubation.

During this process, large amounts of lipids are released being formed specifically by the secretory cells. Therefore, hyphae are sometimes immersed in oil at a later stage of development (Figure 6A). Hyphae encircling the glandular trichomes contain a huge number of globular structures (Figure 6B). Since these structures stain with Nile red, they are likely to be functional equivalents of lipid vesicles detected in *Claviceps purpurea* (Fr.) Tul. harbouring ergoline alkaloids as described by Gröger and his associates [37].

Besides their secretion capacity, the glandular trichomes additionally may provide a site of entry into the plant for metabolites like the alkaloids produced by *Periglandula* species. The transport capabilities of the glandular trichomes were tested using 6(5)carboxyfluorescein diacetate. The uptake of this low molecular weight compound into glandular trichomes and underlying mesophyll cells as well as into fungal cells was observed (Figure 6C,D). However, the stain was not accumulated in epidermal cells of the plant. This indicates that the hydrophobic quality of the cuticle on the epidermal cells restricts uptake of low molecular weight compounds and it may indicate that a secretion of low molecular compounds like ergot alkaloids from the fungal hyphae and specific uptake into the peltate glandular trichomes is likely.

## 3. Discussion

Ergot alkaloids are natural products with an ecological function. This is the reason for an often- encountered symbiosis between an ergot alkaloid producing fungus and a host plant [4,8,15,18,19,22,23,24]. Among these symbiotic associations, the epibiotic clavicipitaceous fungi *P. ipomoeae* and *P. turbinae* are unique since they attach to peltate glandular trichomes on the adaxial leaf surface of their respective dicotyledonous host plants *I. asarifolia* and *T. corymbosa* [8,9,10,11,12,13,17]. A similar system of this type has recently also been described for *Ipomoea carnea* [38]. In this case, an undefined ascomycete socializes with the glandular trichomes of an *Ipomoea carnea* host plant.

We postulate an ergot alkaloid transport within the symbiota *Periglandula*/*Ipomoea* and *Periglandula*/*Turbina*. Similar transport situations were described. Translocation of low molecular weight compounds between an epibiotic nonhaustorial clavicipitaceous fungus (*Myriogenospora atramentosa*) and bahiagrass (*Andropogon virginicus*) has been reported [39]. Opposing leaves of the host were commonly bound together at the tips by bridges of fungal stromata. Sucrose was found to be translocated from one leaf via the fungal stroma to the adjacent leaf. As in our systems, no evidence was seen for a penetration of host tissue by the fungus. 

Transport processes involving pyrrolizidine alkaloids such as loline and *N*-propionylnorloline have also been found [40]. An association between the host grass *Lolium pratense* and its symbiotic endophytic fungus *Epichloë uncinata* (formerly *Neotyphodium uncinatum)* highlights the ecological significance of pyrrolizidine alkaloids and the ecological role of transport processes. The alkaloids do not only benefit the host plant *Lolium pratense* but are occasionally also translocated from the host grass to the hemiparasitic plant *Rhinanthus serotinus*. Loss of the alkaloids from the host plant *Lolium pratense*, and transfer to the hemiparasite, results in a shift from mutualism to parasitism in the grass-fungus relation and benefits the hemiparasitic *R. serotinus* plant in deterring aphids.

We postulate a transport process shifting ergot alkaloids from the *P. ipomoeae* or *P. turbinae* fungi via the peltate glandular trichomes into the *I. asarifolia* or *T. corymbosa* host plant [17]. Microscopic images and the data presented here support this postulate and extend our observations to the *Periglandula*/*Turbina* symbiotum. The system translocating the alkaloids from the epibiotic fungi to the plant is unexplored. Since the biosynthesis of ergot alkaloids is now known, it would be rewarding to investigate the intra- and intercellular transport of ergot alkaloids. This would provide for a deeper understanding of ecological, physiological, and biotechnological aspects of secondary metabolism [41,42]. Transport requires a cellular and morphological differentiation that is triggered by transcription factors [43]. The differentiated plant must be susceptible to the fungus and close contact will result in a plethora of different membrane bound vesicles accumulating at sites of interaction of plants and microbes [44]. Accumulation of vesicles has also been observed in our case (compare Figure 5 and Figure 6), consistent with Gröger’s observation that ergot alkaloid containing vesicles accumulate within the hyphae of *Claviceps purpurea* [37]. Release of ergot alkaloids (clavines and ergopeptines) from a *Claviceps* fungus was noted [45], and the *Periglandula* fungi belonging to the same family Clavicipitaceae may also be able to secrete ergot alkaloids. The close spatial association of the symbiotic organisms as shown above is a prerequisite for a metabolic cooperation. Uptake of alkaloids into plant cells and intercellular distribution may depend on membranes and vesicles, yet an intercellular transport may also proceed through plasmodemata [44,46]. The bidirectional transport may be a feature which is less understood, but it is possible that the two directions of transport occur at different stages of development of the glands [47]. Glandular trichomes of the herbaceous plant *Tanacetum cinerariifolium* (Asteraceae), also known as pyrethrum, is a crop used for the production of insecticidal secondary metabolites collectively called pyrethrins. The biosynthesis of pyrethrins starts within the glandular trichomes and the first biosynthetic intermediate, chrysanthemic acid, is submitted to a basipetal transport within the trichomes. The biosynthesis of pyrethrins is then completed in the pericarp of flower achenes lending immunisation by insecticidal activity to *Tanacetum* embryos and seedlings [47].

A bidirectional transport has also been observed in glands of the carnivorous pitcher plant *Nepenthes alata* [48]. Charles Darwin was the first to describe a bidirectional transport in insectivorous plants after a thorough investigation of glands on the leaves of *Drosera rotundifolia.* Darwin called the glands tentacles and noted “their structure is remarkable, and their functions complex, for they secrete, absorb, and are acted on by various stimulants” [49]. Convolvulaceous glands like those described in this article are often called secretory glands, but we believe, as demonstrated here, that they very likely also have an absorbing function.

The transport systems within the symbiota *Periglandula*/*Ipomoea* and *Periglandula*/*Turbina* discussed here are complex. They involve a sesquiterpene secreting peltate glandular trichome, attachment of an ergot alkaloid delivering clavicipitaceous fungus with an ergot alkaloid secreting function, absorption of ergot alkaloids, and basipetal transport by peltate glandular trichomes, followed by the distribution of ergot alkaloids into the organs of the host plant especially into the ovary securing a seed equipped with ergot alkaloids and completing the lifecycle of the *Periglandula* fungi. Distribution of the alkaloids within the organs of several *Ipomoea* plants is a process that depends on the plant alone. It remains unaffected by the colonizing *Periglandula* fungi [50].

## 4. Experimental Section

### 4.1. Previously Published Materials and Methods

Origin of plant material [8], steam distillation of volatile oil, identification, quantitative estimation (Table 1) and extraction of ergot alkaloids from plant material [13] have been described.

### 4.2. Identification of Terpenoids

The volatile oil was investigated by GC-MS conducted with a Hewlett-Packard HP 5890 gas chromatograph (Boeblingen, Germany) (25 m fused silica capillary coloumn with polydimethylsiloxane CPSil-5, 80 °C starting temperature, 10 °C·min^−1^ temperature program to 230 °C; He as carrier gas) coupled to a VG Analytical 70–250S mass spectrometer (Ringoes, NJ, USA) (ion source temperature 230 °C). Identification of the volatile components was achieved by comparison of their mass spectra and retention indices with a spectral library established under identical experimental conditions [25].

### 4.3. Identification and Quantitative Estimation of Ergot Alkaloids by HPLC/MS/MS

Ergot alkaloids were also qualitatively and quantitatively determined (Figure 3) with an HPLC/MS/MS system using an Agilent series 1100 instrument (Agilent, Palo Alto, CA, USA), equipped with a diode array detector which was set from 275 to 315 nm. Alkaloids were separated by a Nucleodur^®^-PYRAMID C18-column (125 × 2.0 mm^2^, 5 µm) (Macherey-Nagel, Düren, Germany). The column was connected to an API2000 HPLC/MS/MS system (Applied Biosystem/MDS SCIEX, Weiterstadt, Germany) with a Turbo Ion Spray (TIS) as an ion source. Alkaloids were eluted from the column with a solution of 2 mM ammonium acetate in 70% H_2_O mixed with a solution of 2 mM ammonium acetate in 30% MeOH. The mixture was kept constant for 4 min increasing to 100% methanolic solution within 20 min. The flow rate was kept constant at 0.25 mL·min^−1^. The entire system was controlled by the Analyst 1.3 software (Applied Biosystems, Weiterstadt, Germany). Injection volumes were 20 µL of methanolic plant extract or 10 µL of standard material containing authentic ergot alkaloids.

### 4.4. Quantitative Estimation of Ergosterol as a Measure of Fungal Mycelium

This method is based on a procedure published by Schwadorf und Mueller [34]. Freshly harvested leaves of *I. asarifolia* (9 g) or *T. corymbosa* (5 g) were vortexed during 10 min in the presence of a mixture of MeOH (37 mL) and EtOH (25 mL) containing KOH (5 g). The homogenate was submitted to ultrasonic treatment (Sonifier type 250, Branson, Danbury, USA, set to stage 5 and 50% intensity) with 20 pulses (15 s each) interrupted by 15 sec intervals. The homogenate was cooled with ice cubes during sonification. Subsequently, the mixture was heated under reflux at 80 °C for 30 min. After cooling, the reaction mixture was centrifuged (10,000 rpm at 5 °C). The supernatant was extracted two times with petroleum ether. The combined extracts were dried (Na_2_SO_4_), the solvent removed in a rotary evaporator, and the residue dissolved in MeOH (500 µL). The methanolic solution was filtered (membrane filter13 cm diameter, 0.2 µm). Ergosterol was determined quantitatively using a HPLC system (Hitachi, Tokyo, Japan) equipped with a L-4000 UV detector set to 282nm and a 5 µm Nucleodur^®^ C_18_ column (250 × 4 mm) (Macherey-Nagel, Dueren, Germany). The molar absorption coefficient of ergosterol is ε = 11,900 at 282 nm.

### 4.5. Production and Characterisation of a Polyclonal Antibody against the 4-(γ,γ -Dimethylallyl) Tryptophan Synthase (DmaW) from P. ipomoeae

**Figure 7:**
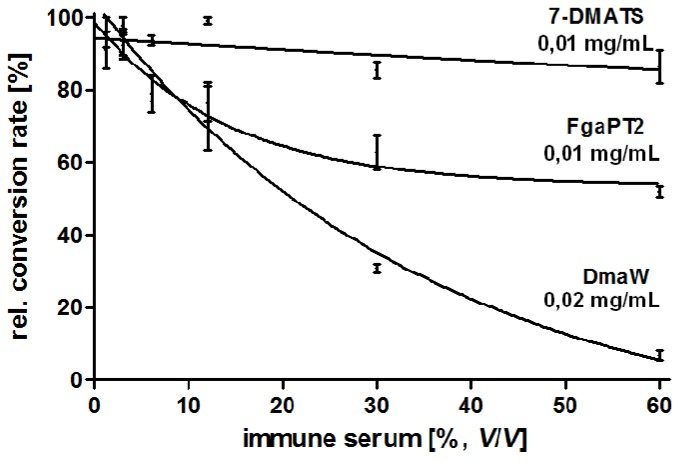
Specificity of a polyclonal antibody directed against the His-tagged 4-(γ,γ-dimethylallyl)tryptophan synthase (DmaW) from *P. ipomoeae*. The specificity against corresponding tryptophan prenylating enzymes (FgaPT2 and 7-DMATS) from *A. fumigatus* is significantly lower. The amount of DmaW enzyme from *P. ipomoeae* was doubled when compared to FgaPT2 and 7-DMATS.

Production and purification of the recombinant His_6_-tagged DmaW enzyme was previously described [17]. Seqlab Sequence Laboratories Göttingen GmbH, Germany, was commissioned to produce rabbit immuno serum containing antibodies against His_6_-DmaW by using three injections of 200 µg enzyme in 50 mM Tris-HCl pH 7, and complete five in a two month protocol. Preimmune serum was also taken and used as a control. The specificity of the resulting antibody was tested against His-tagged DmaW from *P. ipomoeae* and two related His-tagged tryptophan prenylating enzymes from *Aspergillus fumigatus*, such as 4-(γ,γ-dimethylallyl)tryptophan synthase (FgaPT2) and 7-(γ,γ*-*dimethylallyl)tryptophan synthase (7-DMATS). The two latter enzymes were prepared as described for FgaPT2 [51] and for 7-DMATS [52,53]. The prenylation reaction of all three tryptophan prenylating enzymes was now tested (Figure 7) in the presence of anti-DmaW serum using enzyme concentrations of 0.01 mg/mL 7-DMATS, 0.01 mg/mL FgaPT2 but 0.02 mg/mL DmaW, each calculated as monomer and solved in 90 µL 50 mM Tris-HCl pH 7,5 and of anti-DmaW serum with increasing concentrations. Incubation was carried out for one hour at 30 °C. The samples were then centrifuged for 30 min at 13.000× g and 4 °C. The supernatant was transferred into a new tube. After addition of 1 mM L-tryptophan, 1 mM DMAPP (dimethylallyl diphosphate), and 5 mM CaCl_2_, the assays were incubated at 30 °C for one hour. The enzymatic reaction was then stopped with 12.5 µL TCA (trichloroacetic acid, 1.5 M). The samples were analysed by HPLC [17]. As expected, the highest specificity of the antibody was observed for the DmaW enzyme from *P. ipomoeae* when compared to those from *A. fumigatus* (Figure 7).

### 4.6. Microscopic Techniques

Light microscopy was carried out with a Leitz DMR photomicroscope equipped with Normaski interference contrast and epifluorescence (Leica, Bensheim, Germany). For image processing, a digital camera KY-F75 and Diskus^®^ software (TB Hilgers, Königswinter, Germany) was used. Fungal structures on the leaf surface were stained as described [8,13]. Visualization of alkaloids in the epiphytic mycelium by autofluorescence was done according to Mulac *et al.* [33]. 6(5)carboxyfluorescein diacetate (Sigma-Aldrich, St. Louis, MO, USA) was used at an excitation wavelength of 450 to 490 nm for the uptake assays with 6 × 10^−5^ in 0.5 M sucrose solution.

Leaf samples were fixed in 2.5% glutaraldehyde and 2% paraformaldehyd in 0.1 M sodium cacodylate, pH 7.3 for 24 h [54]. The tissue was dehydrated and embedded in L.R. White acrylic embedding medium (Polysciences Inc. Warrington, PA, USA). Semi-thin sections were cut with a glass knife and stained with toluidine blue (0.1% aqueous). For transmission electron microscopy (TEM) performed with an EM109 (Carl Zeiss, Jena, Germany), samples were fixed and cut with a diamond knife according to Schumacher *et al.* [55].

For scanning electron microscopy (SEM) performed with a XL 30 SFEG (Philips, Eindhoven, The Netherlands), the samples were critical-point dried from CO_2_ in eight cycles after dehydration according to Svitkina *et al.* [56] with a Balzer CPD 030 (BAL-TEC, Schalksmühlen, Germany). Dried specimens were mounted on aluminium sample holders and sputter coated with 2 nm platinum/palladium with a HR 208 coating device (Cressington, Watford, UK). SEM was performed with a XL 30 SFEG (Philips, Eindhoven, The Netherlands).

The tissue print immunopressblotting with the antibodies against His_6_-DmaW was done according to Quadt-Hallmann *et al.* [57]. Leaves colonised by the fungi were placed on a nitrocellulose membrane and press blotted between flat pieces of iron for 3 minutes. All reactions were performed at room temperature. Blocking of unspecific protein-binding sites was carried out by incubation in PBS, pH 7.4 with 2% PVP, and 1% BSA. The blot was rinsed in buffer (PBS, pH 7.4 and 0.05% Tween 20) three times for 15 min each and incubated with antibodies, conjugated to alkaline phosphatase for 5 h. After washing, the blot was transferred to the substrate Nitro tetrazolium chloride (NBT)-solution and 5-bromo-4-chloro-3-indoxyl phosphate (BCIP)-solution. Blue colour development was stopped by rinsing several times with distilled water and 0.02 mol/L Tris/HCl buffer. Micrographs were taken under a stereomicroscope MZ 16F (Leica, Bensheim, Germany) equipped with a digital camera KY-F75 and Diskus^®^ software (TB Hilgers, Königswinter, Germany) for image processing.

## 5. Conclusions

We describe observations that peltate glandular trichomes on the adaxial leaf surface of two Convolvulaceae, *I. asarifolia* and *T. corymbosa* and their epibiotic clavicipitaceous fungi, *P. ipomoeae* and *P. turbinae* respectively form functional units. While the two fungal species never penetrate the leaf epidermis, their hyphae are intimately associated with the glandular trichomes of their host plants and are even detectable underneath the cuticle of trichomous cells. The fact that ergot alkaloids are synthesized within the hyphae but sequestered in the leaves led to the conclusion that the glandular trichomes absorb and translocate the alkaloids into the leaf lamina. Since the alkaloids and the producing fungi are vertically seed transmitted, the leaves are not the terminal site of deposition; rather, alkaloids are forwarded to the flowers and possibly also to other plant organs. These latter processes are solely plant dependent. The two symbiotic systems discussed here offer an opportunity to further investigate function, physiology, and transport processes that take place within the peltate glandular trichomes and in convolvulaceous host plants.
